# Predicting Long-Term Endothelial Cell Loss after Preloaded Descemet Membrane Endothelial Keratoplasty in Fuchs’ Endothelial Corneal Dystrophy: A Mathematical Model

**DOI:** 10.3390/jcm13030877

**Published:** 2024-02-02

**Authors:** Pietro Viola, Enrico Neri, Tommaso Occhipinti, Mohit Parekh, Roberto Cian, Diego Ponzin, Antonio Moramarco, Alfonso Iovieno

**Affiliations:** 1 Ophthalmology Unit, San Bortolo Hospital, 36100 Vicenza, Italy; dr.pietro.viola@gmail.com (P.V.);; 2 Department of Ophthalmology, Schepens Eye Research Institute of Mass Eye and Ear, Harvard Medical School, Boston, MA 02115, USA; mnparekh@meei.harvard.edu; 3 Fondazione Banca degli Occhi del Veneto Onlus, 30174 Venice, Italy; 4 Ophthalmology Unit, IRCCS, Azienda Ospedaliero-Universitaria, 40138 Bologna, Italy; 5 Department of Ophthalmology and Visual Sciences, University of British Columbia, Vancouver, BC V5Z 1L3, Canada

**Keywords:** DMEK, preloaded, FECD, long term, endothelial cell loss, mathematical model, prediction

## Abstract

(1) **Background**: This study offers a biexponential model to estimate corneal endothelial cell decay (ECD) following preloaded “endothelium-in” Descemet membrane endothelial keratoplasty (DMEK) in Fuchs’ endothelial corneal dystrophy (FECD) patients; (2) **Methods**: A total of 65 eyes undergoing DMEK alone or combined with cataract surgery were evaluated. The follow-up period was divided into an early phase (first 6 months) and a late phase (up to 36 months). Endothelial cell count (ECC) and endothelial cell loss (ECL) were analyzed; (3) **Results**: The half time of the ECD was 3.03 months for the early phase and 131.50 months for the late phase. The predicted time-lapse interval to reach 500 cells/mm^2^ was 218 months (18.17 years), while the time-lapse interval to reach 250 cells/mm^2^ was 349 months (29.08 years). There was no statistically significant difference between the ECL in DMEK combined with cataract extraction and DMEK alone at 24 months (*p* ≥ 0.20). At the late phase, long-term ECL prediction revealed a lower ECC half time in patients undergoing DMEK combined with cataract surgery (98.05 months) than DMEK alone (250.32 months); (4) **Conclusions**: Based on the mathematical modeling, a predicted average half-life of a DMEK graft could reach 18 years in FECD. Moreover, combining cataract extraction with DMEK could result in excessive ECL in the long term.

## 1. Introduction

The human cornea is the anterior tissue of the eye that is important to refracting the surrounding light to the retina. Corneal endothelial cells (CECs) underlie the posterior part of a monolayer of hexagonal cells that do not possess a regenerative potential; therefore, they must be preserved to maintain the transparency of the tissue and in turn clear vision. CECs pump the water and ions to and from the cornea to maintain the transparency and thickness of the tissue. Disease or dysfunction of the CECs can potentially lead to the loss of cell numbers and hence accumulation of fluid in the cornea, leading to edema and loss of vision, along with pain. The only available treatment to cure corneal endothelial dysfunction is corneal transplantation [1]. However, due to the limited supply of human donor corneal tissues, the treatment is significantly restricted. It therefore becomes important to also ensure that these tissues survive for a long term. 

Endothelial cell decay has been extensively studied in various ocular disorders and after surgical procedures like full-thickness corneal transplantation [1]. Endothelial cell count (ECC) is considered the main determinant of graft survival after keratoplasty [2,3,4]. A mathematical biexponential model has been used to describe endothelial cell decay, both in healthy eyes and after different types of eye surgeries [5]. The biexponential curve evidences two different phases: a rapid component in the early postoperative period (the first 6 months) followed by a slow component. This model has been previously applied in penetrating keratoplasty (PKP) and Descemet’s stripping automated endothelial keratoplasty (DSAEK) [5,6]. Descemet membrane endothelial keratoplasty (DMEK) is a relatively new surgical technique for selective transplantation of the Descemet membrane and the endothelium [7]. This technique has achieved great success since its introduction by Melles et al. [8] in 2006, and its attractiveness is increasing worldwide due to its rapid visual recovery and optimal postoperative visual acuity [9]. The main indication for DMEK is Fuchs’ endothelial corneal dystrophy (FECD), which is the most common corneal endothelial dystrophy [10] and the main indication for corneal transplantation worldwide [11].

Fuchs’ endothelial corneal dystrophy is recognized as a complex and heterogeneous genetic disease with variable expressivity and incomplete penetrance. It affects both eyes with a slowly progressive course, appearing around the age of 30–40 years and impairing vision generally after the age of 50 years, with a higher incidence in women than men, with a ratio of 3–4:1 [12,13,14,15]. Female sex and age are the most significant risk factors for advanced FECD development [15], but there are also additional risk factors, such as family history, smoking, and diabetes; we thus recognize a multifactorial etiology where genetic and environmental factors interact [16,17,18]. Fuchs’ endothelial corneal dystrophy is characterized by the progressive decline of corneal endothelial cells, leading to apoptosis, variations in the size (polymegeticism) and shape (pleomorphism) of corneal endothelial cell morphology, a decrease in endothelial cell density, and the formation of extracellular matrix outgrowths called guttae [14,19,20,21] mainly in the central cornea [22]; due to endothelial decompensation, edema is formed, and the cornea loses its transparency; there is marked reduction in vision acuity, and acute pain may occur due to the rupture of epithelial bubbles.

There are few studies to date reporting long-term follow-up after DMEK [23,24]. However, predicting a long-term ECL following DMEK surgery using a mathematical model is not available. In addition, preloaded DMEK is a relatively new technique that allows a ready-to-use prevalidated graft prepared by the eye bank [25], which can be delivered either using the endothelium inwards [26,27,28,29] or outwards method [30,31,32] and has a potential to be shipped internationally [33,34,35]. As this technique is emerging as a standard of care in our tertiary center, we further investigated the long-term ECL predictability of these grafts. Hence, the purpose of this study is to present a mathematical biexponential model to determine the long-term endothelial cell decay after DMEK surgery.

## 2. Materials and Methods

### 2.1. Inclusion Criteria and the Ethical Statement

This was a single center study. Sixty-five eyes of sixty-five patients (31 males and 34 females) aged between 57 and 92 years (mean patient age was 73.2 ± 7.5 years) affected by FECD were included in this retrospective study. Patients with any ongoing eye disease or with a history of ocular surgery (apart from cataract surgery) were excluded.

DMEK was performed by a single surgeon (PV) as a standalone procedure or in association with cataract extraction and IOL implantation.

This study was conducted in accordance with the Helsinki Declaration of the World Medical Association, and it was approved by the local institutional review board. Written informed consent was obtained from all the patients. The Ethics Committee waived the need for ethics approval and the need to obtain consent for the collection, analysis, and publication of the retrospectively obtained and anonymized data for this retrospective study. 

### 2.2. DMEK Graft Preparation and Surgery

Preloaded donor corneas deemed suitable for transplantation were obtained from the Veneto Eye Bank Foundation (Venice, Italy) with written consent from the donor’s next-of-kin [26]. Briefly, the tissues were analyzed for suitability in the eye bank and washed in sterile PBS to remove any media remnants. The corneas were mounted with the endothelial side facing the air, on a base, and stained with trypan blue (Vision blue) for 30 s followed by washing with sterile PBS. The peripheral endothelium was scored using a Sinskey hook along the entire 360° circumference, and the Descemet membrane–endothelial complex was stripped with an acute forceps (non-toothed) from the inferior to the superior position leaving a small peripheral hinge behind. The stripped tissue was re-stored back on the stroma using a gentle flow of PBS after punching the stroma with a 2 mm biopsy punch. The tissue was inverted (epithelium facing the air) and marked with an “F” stamp on the Descemet membrane using a skin marker containing gentian violet. The tissue was reinverted with the endothelium facing the air and successively punched to a diameter of 8.25 mm [26,28,36,37]. Grafts were then preloaded with the endothelium tri-folded inwards in a 2.2 mm intraocular lens (IOL) cartridge (Viscoject, Wolfhalden, Switzerland). Preloaded corneal grafts were placed into a dedicated sterile vial and positioned into a specific container [38]. All grafts were delivered and utilized within 24 h from preparation. DMEK was performed by a “pull-through” technique [39]. Grafts were stained with 0.06% VisionBlue solution (DORC, Zuidland, The Netherlands) intraoperatively. Inferior basal iridotomy was also performed intraoperatively.

### 2.3. Preoperative and Postoperative Analysis

All eyes were routinely examined preoperatively and postoperatively at 1, 3, 6, 9, 12, 18, and 24 months. Fourteen eyes were also examined at 36 months. Central noncontact specular microscopy (EM-3000, Tomey, Phoenix, AZ, USA) was used for endothelial cell count (ECC, cells/mm^2^). Endothelial cell loss (ECL, %) was calculated based on the preoperative donor corneal ECC (provided by the eye bank) and postoperative ECC at each time interval.

### 2.4. Mathematical Model

Mathematical modeling was performed using Microsoft Excel (version 16.44) and the MATLAB (Version R2023b, Mathworks, Natick, MA, USA) system. Based on previous publications, two components of postsurgical ECL can be defined as an early rapid phase (until the 6th month postoperative) and a late slow phase (beyond 6 months postoperative). In order to describe endothelial cell decay over time in a mathematical function, we analyzed all different monoexponential and biexponential functions in relationship to the average values of ECC over 24 months obtained from our patients. The parameters of this model were calculated in MATLAB using a nonlinear least squares algorithm. Goodness of fitting was expressed by an R-square value (range between 0 and 1, with a value closer to 1 indicating a greater proportion of variance accounted for by the model). Once the best fitting curve and corresponding equation had been identified, the curve was extended over time to obtain our prediction model. The equation of the model was specified as follows: Fit (x) = a × exp (b × x) + c × exp (d × x), where x represents the time (in months). For each exponential term, a (or c) represents a scaling factor of the exponential function, whereas b (or d) is the power of the decrease (negative value) or of the increase (positive value); a (c) scales the value of the fit to the range of the values that were observed, b (d) provides the “speed” of the decrease in the two phases (early/late) in time. Half times for the early phase and late phase of the decay were calculated as ln 2/exponential rate constant (that is, “b” for the early phase and “d” for the late phase) [5,6].

To assume that both the reduced model curves (“with phaco” and “without phaco” groups) have a best fitting curve comparable with the full data group, a likelihood ratio test (LRT) was used. The two LRTs have been computed by using the MATLAB function “lratiotest” imposing an output result “h” (logical value 0 or 1). By this function, a result of h = 1 indicates that the null, restricted model should be rejected in favor of the alternative, unrestricted model. In the case of h = 0, the null hypothesis should be accepted (the reduced model curves are comparable to the full data model). A chi-squared test was used to determine the difference in rebubbling rate between patients with and without associated cataract surgery. Continuous data were analyzed using the Student’s *t*-test for differences between the groups.

## 3. Results

**Figure 1:**
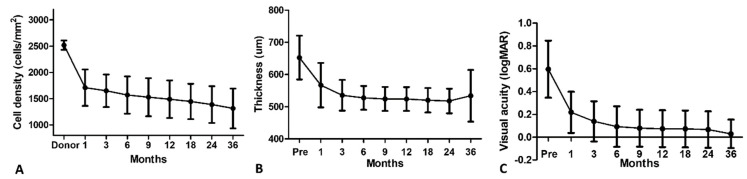
Outcomes of (**A**) endothelial cell density, (**B**) central corneal thickness, and (**C**) visual acuity measurements over a 36-month time period.

Sixty-five eyes of sixty-five patients (31 males, 34 females) underwent DMEK surgery. The mean donor age was 66.9 ± 6.8 years with a male–female ratio of 29:36. The mean postmortem interval was 12.0 ± 6.9 h. The tissues were preserved for 10.8 ± 3.8 days in organ culture media and 3.0 ± 1.1 days in the transport media in the eye bank before surgical use. The preoperative ECC of the donor graft was 2518.46 ± 89.95 cell/mm^2^ (range: 2300–2700 cells/mm^2^). The mean patient age was 73.2 ± 7.5 years (range: 57–92 years). Phaco was performed in 35/65 (53.8%) cases. Moreover, 21/65 (32.3%) cases required one rebubbling, and 2/65 (3%) cases required two rebubbling events, mostly within the first two weeks of the surgery. Endothelial cell density, BCVA, and central corneal thickness measurements over time are shown in Figure 1.

**Figure 2:**
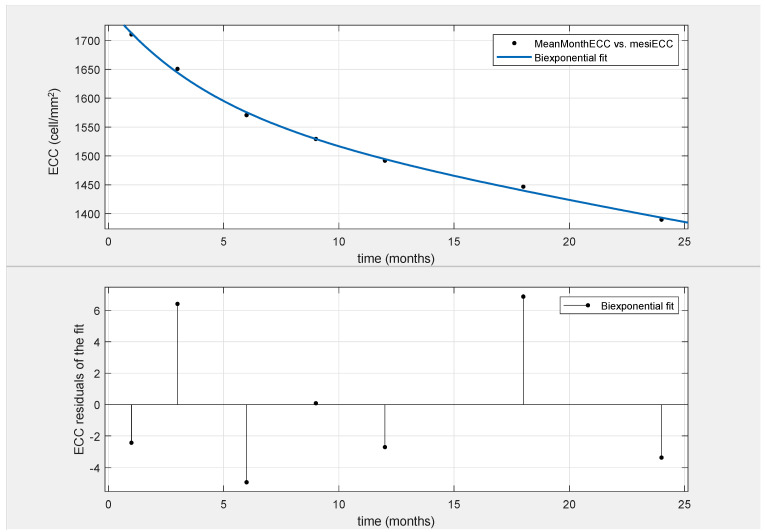
Fitting curve (continuous line) related to the ECC mean value over the first 24 months (**top** figure) and residual plot showing goodness of model fitting (**bottom** figure).

By analyzing endothelial cell decay in our series of patients, a second-order exponential decay function was found to be the best model to express ECC over time in DMEK (Fit (x) = a × exp (b × x) + c × exp (d × x); R square = 0.9982; Figure 2). The four parameters of the equation were a = 177.5; b = −0.2298; c = 1580; and d = −0.005271.

**Figure 3:**
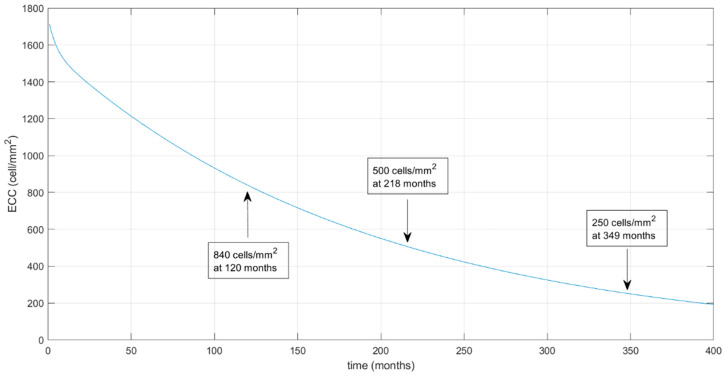
Prediction model biexponential curve extended over time. The two arrows indicate mean ECC values of 500 cells/mm^2^ (at 218th month) and 250 cells/mm^2^ (at 349th month). ECC: mean endothelial cell count (cells/mm^2^).

The extrapolated prediction model with an extension of the curve over time until the ECC reaches the predicted values of 500 and 250 cell/mm^2^ (which occurred at 218 and 349 months, respectively) is shown in Figure 3.

The endothelial cell decay half time was 3.03 months in the early phase and was found to be 131.50 months in the late phase. Using our model, the predicted ECL for DMEK was 48% at 3 years, 54% at 5 years, and 67% at 10 years.

**Figure 4:**
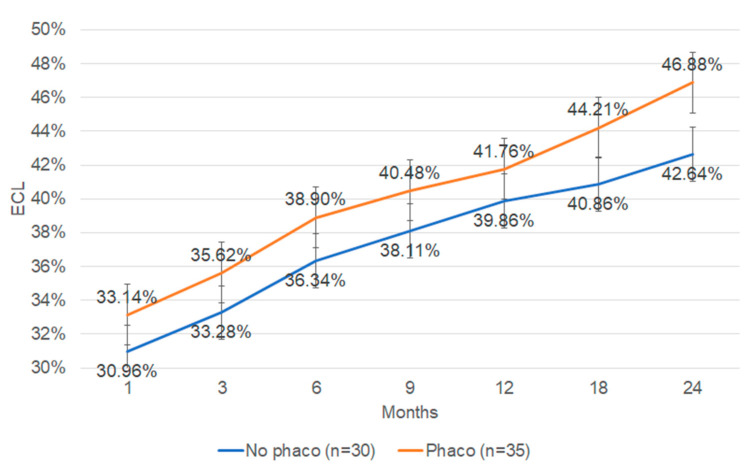
ECL in DMEK combined with cataract surgery vs. DMEK without cataract surgery. ECL: endothelial cell loss; DMEK: Descemet membrane endothelial keratoplasty. Maximum *p* value is 0.58 at 12 months. Minimum *p* value is 0.20 at 24 months.

The effect of cataract extraction combined with DMEK on ECL was further analyzed. Thirty-five eyes (53.85%) underwent DMEK combined with cataract extraction and IOL implantation. Thirty eyes (46.15%) underwent DMEK surgery alone. In both cases, the h value equaled 0. The ECL in DMEKs with or without cataract surgery in our cohort of patients did not show any statistical difference between the groups at any time point over 24 months (Figure 4).

**Figure 5:**
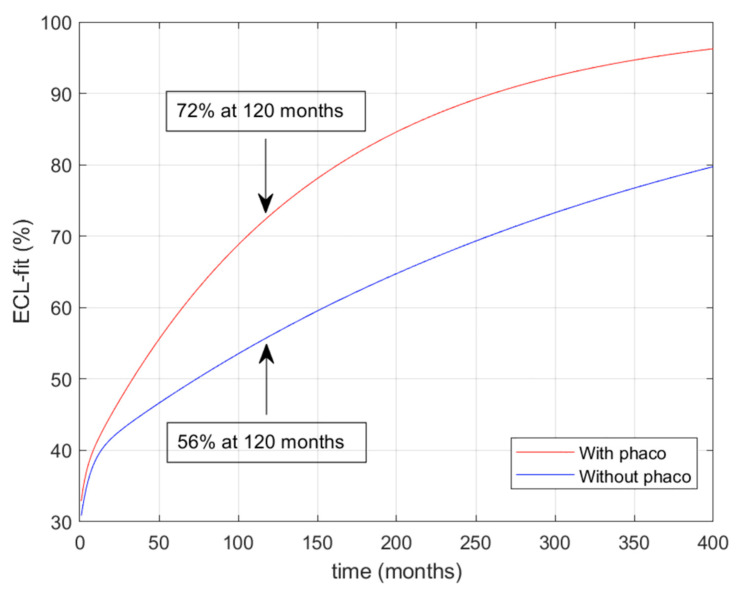
Biexponential functions of ECL over time in DMEK with (with phaco line, in red) and without (without phaco line, in blue) cataract surgery. (ECL: endothelial cell loss.)

The ECL prediction over time in both groups is represented in Figure 5. It reaches 72% at 120 months postoperatively in the group that underwent cataract surgery (red line in figure). In the group with only the DMEK procedure, the ECL prediction is 56% at 120 months from surgery (blue line in figure).

The early phase endothelial cell decay half time was 2.26 months in the DMEK-only group and 4.06 in the DMEK with phacoemulsification group, respectively; in the late phase, the half time was 98.05 and 250.32 months, respectively (not shown in the figure).

## 4. Discussion

Descemet membrane endothelial keratoplasty is a variant of endothelial keratoplasty for the treatment of corneal endothelial dysfunction. According to an analysis conducted by the American Academy of Ophthalmology (AAO) in 2017, the number of DMEK procedures performed over the past 10 years in America has steadily increased in parallel with the reduction in the number of Descemet’s stripping endothelial keratoplasty (DSEK) procedures [40]. The increase in the number of DMEK procedures appears to coincide with the ability of eye banks to offer preprepared DMEK tissue, the rapid growth of the scientific literature, and numerous DMEK training and skills transfer courses. In the analysis conducted by the AAO [40], despite the difficult learning curve and rebubbling rates observed with all types of DMEK procedures, including surgeon-prepared or eye-bank- prepared (prestripped or preloaded) tissues, there appears to be sufficient evidence to show that DMEK is superior to DSEK in achieving faster visual recovery, better visual outcome, and a lower immune rejection rate. Evidence also suggests that DMEK induces a lower refractive error than DSEK. The rate of ECL, the primary and secondary transplant failure rates, and complications during and after DMEK are comparable to those during and after DSEK, which has been considered to be a gold standard for endothelial keratoplasty in the past decade [40].

The surgical practice of endothelial keratoplasty was first described in 1956 as “Posterior Lamellar Keratoplasty” [41]. Since then, the technique has evolved rapidly and has been refined: manual dissection of the posterior stroma has been replaced by removal of the Descemet membrane (DM) from the recipient (descemetorhexis), and manual dissection of the donor tissue has been replaced by the use of the automated microkeratome [42,43]. This surgical procedure is called “automated endothelial keratoplasty with Descemet’s stripping” and is referred to as DSEK. The concept of DMEK was introduced in 2002, and the first-use case of DMEK was published in 2006 [8]. In DSEK, a thin layer of the posterior stroma, the Descemet membrane, and the endothelium are transplanted as a single lamellar graft onto the surface of the posterior stroma of the host after the descemetorhexis phase. In contrast, in DMEK, only the endothelium and the Descemet membrane are transplanted, and this eliminates the problems of interface irregularity and the refractive defect due to the uneven stromal thickness of the microkeratome-cut DSEK graft.

The current technique involves the lenticule of the endothelium and the Descemet membrane being separated from the donor corneal stroma, rolled up with the endothelium facing outwards, and loaded into the intraocular delivery device. At this point, it is ready to be stained with Trypan blue.

After manually removing the central DM from the recipient (descemetorexis) under air, cohesive viscoelastic, or fluid, the graft is introduced into the anterior chamber using an injector. The graft roll is deployed by the operator using techniques that include a light tapping on the cornea (no-touch technique) [44], a combination of small sprays of balanced saline and air under the endothelium [45,46], or a light rolling on the corneal surface [21,22,23,24,25,26,27,28,29,30,31,32,33,34,35,38,39,40,41,42,43,44]. The correct orientation of the graft is confirmed by orientation marks on the edge of the DM graft [47], by a blue cannula mark [44], by ink marks on the DM side of the graft [48], or by the use of optical coherence tomography (OCT) during surgery [49]. As soon as the donor tissue is unfolded in the correct orientation and centered, air or sulphur hexafluoride gas (SF6; 14% or 20%) is injected underneath the graft to fix it to the recipient cornea. The air or gas injected into the anterior chamber can cause pupillary blockage, which is why some surgeons remove a small amount of it at the end of the procedure, while others prefer to perform peripheral iridectomy before or during the DMEK procedure. After the operation, the patient will have to lie supine for 1–3 days to allow for the maximum buffering effect of the air or gas.

The main complications of DMEK include graft detachment, graft failure, elevated intraocular pressure (IOP), immune rejection, cystoid macular edema (CME), and endothelial cell loss (ECL).

The most common complication of DMEK is graft detachment, which can vary in size. Small peripheral detachments may reattach spontaneously without affecting the final visual outcome [50,51], whereas large detachments or detachments involving the visual axis require reoperation (rebubble or new DMEK). To predict in which eyes a graft detachment may or may not occur, Patefield et al. recently developed a new accurate algorithm using multiple-instance learning artificial intelligence (MIL-AI) based on pre-Descemet membrane endothelial keratoplasty (DMEK) anterior segment optical coherence tomography (AS-OCT) images [52].

The second most common complication after DMEK surgery is IOP increasing (an increase of ≥10 mmHg compared to preoperative IOP or values >24 mmHg). Angle closure or pupillary blockage may lead to increased IOP during the immediate postoperative period, but in most cases, the increase in IOP after DMEK is induced by postoperative corticosteroid therapy [53,54].

Regarding transplant rejection, the average rejection rate during follow-up periods from 6 months to 8 years decreases progressively [55], and it is close to 0% in patients who continue low-dose topical corticosteroids during the first year after surgery, while it is 6% in patients who suspended topical corticosteroids earlier [56].

Cystoid macular edema (CME) is another complication after DMEK. In most cases, the rate of CME development is less than 7.4%, with a higher frequency after DMEK combined with cataract surgery than after the single DMEK procedure [57]. One study showed that intensive topical corticosteroid treatment during the first week after surgery reduced the CME rate from 12% to 0% after combined DMEK and cataract surgery [57].

Additionally, endothelial cell loss (ECL) is among the complications of DMEK surgery too. According to the analysis carried out by AAO [40], a significant decrease in average EC density (27–46%) occurs in the first 3 months postsurgery, and the level of reduction gradually decreases thereafter. At 6 months, the average loss of EC is 33% (25–47%). Three studies [58,59,60] reported a loss of EC of 42% at 4 years, 39% at 5 years, and 65% at 7 years, respectively. Air injection [58], the use of SF6 [46], and graft marking [48] have been shown not to increase ECL. Intense topical corticosteroid treatment during the first week following DMEK surgery had no effect on ECL at 6 months [61]. Eyes with previous trabeculectomy or shunt for glaucoma have greater ECL than those without [62].

It is currently not possible to accurately predict a long-term ECL following DMEK surgery. The purpose of this study is to present a mathematical biexponential model to determine the long-term endothelial cell decay after DMEK surgery.

Biexponential regression has been chosen as the most suitable model for predicting ECL after DMEK [5,6]. The R-Square index is a parameter that describes how much endothelial cell decay over time can be fitted in each kind of mathematical function. The more the R-Square index is close to 1, the better the mathematical function describes endothelial cell decay. Such a function has never been used to identify the ECL after DMEK and to predict the long-term ECL. Amongst all single and double mathematical functions, the biexponential regression R-Square index was the closest to 1 (values for other models were not shown in the text).

However, there is a limited literature related to developing prediction models of ECL after keratoplasty [4,5,6]. To the best of our knowledge, only penetrating keratoplasty (PKP) and Descemet’s stripping (automated) keratoplasty (DSEK/DSAEK) have been analyzed to date. Endothelial cell decay after keratoplasty is usually considered to occur in two phases, an early and a late phase. In the early phase (usually less than 6 months), the half-life was 3.03 months. This value is comparable to the studies conducted by Dooren et al. on DSAEK [7], where the early-phase half-life was 2.2 months overall, with no significant effect of pseudophakic bullous keratopathy (PBK) or simultaneous FECD and PBK [6]. These data confirm that the early ECC decline pattern in DMEK is similar to that of DSAEK and different from that of PKP, where early ECC half-life is longer (12.8 months) [6].

Simultaneously, we also encountered a difference in the late-phase half-life in comparison to a previous DSAEK model of ECC (131.50 months in our study vs. 75.5 months in Dooren’s study [6]). DMEK may therefore potentially result in a longer survival rate compared to DSAEK. It has been hypothesized that donor endothelial cell redistribution onto the recipient cornea could be responsible for late endothelial failure of the graft [63]. The additive stromal component of a DSAEK graft could represent a physical barrier to donor endothelial cell migration onto the recipient, hence hampering the redistribution. The lack of a stromal component in DMEK surgery may facilitate cell migration and reduce late-phase endothelial count. The findings of our study do not seem to support this hypothesis. Our results are corroborated by comparing our data with the 10-year retrospective series, conducted by Melles’ group [23]. In this study, an average 10-year ECC was 903 ± 356 cells/mm^2^, which is remarkably similar to our modelled outcome (840 cells/mm^2^ at 10 years). The slope of the curves in the two studies also shows a high degree of similarity. In general, corneal grafts are thought to remain clear until the ECC declines to a value of 250 to 500 cells/mm^2^ [64,65,66,67]. Using our estimated model, the time-lapse in which ECC would decrease to 500 cells/mm^2^ would be 18 years.

Cataract surgery combined with DMEK resulted in additional ECL over time; however, no significant difference in ECL was found in the first 24 months postoperative. In our model, ECL at 120 months was 56% in the DMEK-only group and 72% in the DMEK combined with cataract extraction group, respectively. Whether combined cataract surgery produces additional ECL in DMEK surgery remains a controversial issue. Fajardo-Sanchez et al. reported that performing phacoemulsification before DMEK or opting for a combined procedure did not seem to affect the survival rate up to 1 year postoperative [68]. This conclusion is in contrast to Shahnazaryan et al., where combined DMEK–cataract surgery resulted in significantly greater loss of endothelial cells than DMEK-only at both 1 month and 1 year postoperative [69].

The sudden anterior chamber deepening following cataract surgery has been considered as a main factor that could negatively influence the postoperative ECC, but it was thought to alter mainly the early phase ECC than the late phase ECC [6]. Excessive ECL in combined cataract cases could also be due to fibrin release at the time of cataract extraction. The prolonged surgical time along with the pupillary dilation followed by miosis could determine fibrin release in the anterior chamber, resulting in longer graft manipulation, increased surgical time, and ultimately excessive ECL [69].

The limitations of this study include the homogeneity of the donors and patients. However, the real-time scenario, as shown in this study, best represents the surgical outcome; therefore, our model would be suitable for a general population, if not a specific one. Our data included a 32% rebubbling rate and 53% phaco cases, which are still among the concerns following DMEK or triple procedures that are observed widely. Therefore, including these criteria only makes the model reliable for the general population. Although we only modeled the average endothelial cell loss over time, a survival analysis to model the time until a certain threshold of cell loss is reached would be of interest in the future.

Surgical experience could possibly be considered a cofounder in our study. Prior to the cases included in this study, the surgeon (PV) had performed 30 DMEK surgeries with different techniques and five previous preloaded endothelium-in DMEKs. Those early cases were excluded to reduce the potential bias introduced by the learning curve [55]. A learning effect has already been described as one of the main factors that could influence early-phase ECL [6]. On the other hand, in a single-surgeon series, the first 250 eyes undergoing DMEK surgery have shown a lower survival probability than the following 250 eyes [70,71]. A confounding effect of surgical experience on the results of our study could therefore be a potential limitation.

We believe that the presented mathematical model for the prediction of endothelial cell loss following DMEK represents by and large a general cohort of DMEK patients and therefore would be a relevant method to predict ECL. In the near future, analyses of longer-term results based on higher numbers of patients will further confirm the accuracy of our ECL prediction model in DMEK.

## Data Availability

The original contributions presented in the study are included in the article, further inquiries can be directed to the corresponding author.
